# Editorial: Leveraging pharmacovigilance data mining with “the patient” in mind

**DOI:** 10.3389/fphar.2022.980645

**Published:** 2022-08-15

**Authors:** Maxine Gossell-Williams, Maribel Salas

**Affiliations:** ^1^ Section of Pharmacology and Pharmacy, Faculty of Medical Sciences Teaching and Research Complex, University of the West Indies, Kingston, Jamaica; ^2^ Clinical Safety and Pharmacovigilance, Daiichi Sankyo, Inc., Basking Ridge, NJ, United States; ^3^ Center for Real-world Effectiveness and Safety of Therapeutics (CREST), University of Pennsylvania Perelman School of Medicine, Philadelphia, PA, United States

**Keywords:** pharmacovigilance, individual case safety report, patient, adverse events, pharmaceutical

Despite the emergence of new technologies, the use of individual case safety report (ICSR) is a central activity in the advance of pharmacovigilance. Revisions in causality assessment continue to be a topic of interest, such as the recently reported vigiGroup clustering system (Norén et al., 2021). Furthermore, some groups are working on the data integration through artificial intelligence, particularly machine learning (Kreimeyer et al., 2021; Ball and Dal Pan, 2022). However, while the objectives of these advances is fundamental to improve the signal detection process, the eleven papers in this Research Topic serve as reminders that the multifactorial nature of “the patient” must remain at the forefront of any pharmacovigilance-related activities, including detection, understanding, assessing, preventing, managing and communicating adverse drug events.

As part of pharmacovigilance activities, data mining of adverse events related to pharmaceuticals is critical for early identification of potential safety signals, validation of safety signals, risk assessment and establishment of risk minimization activities. Databases to support the collection of ICSR may be used in early identification monitoring systems for specific adverse events, such as the National Tuberculosis Program in Pakistan which Massud et al. reported as minimizing the risk of adverse events among drug-resistant *tuberculosis* patients. However, most adverse event patterns for signal detection are gained through wider scope systems, such as those utilized by five articles appearing in this Research Topic (Guo et al.; Ji et al.; Liu et al.; Khan et al.; Cai et al.).

The importance of ICSR databases for previously undescribed safety concerns was highlighted by Khan et al., where ICSR from the pharmacovigilance center of the Balcali Hospital in Turkey were used to explore differences in adverse event profiles across pediatric age groups; a population which the authors reported have limited available data in that country. Guo et al. using the Henan Adverse Drug Reaction Monitoring Center in China determined signals for adverse events not previously described for antipsychotic drugs. Liu et al. used the US Food and Drug Administration Adverse Event Reporting System, one of the largest national safety databases, to examine known cardiotoxic adverse events of anaplastic lymphoma kinase tyrosine inhibitors and identified difference across the drug class.

The incongruence between frequentist and Bayesian disproportionality methodologies of signal detection was discussed by Liu et al. and has been supported by other authors (Faich and Morris, 2012; Lee et al., 2020; Khouri et al., 2021). Ji et al. explored an analytical modeling that integrates pharmacology information, such as known drug adverse event associations and other intrinsic drug properties with Bayesian disproportionality analytics. The modeling advanced by these authors is likely to stimulate conversations on a hopeful transformational improvement.

The relevance of phenotypic and genetic information in the adverse events process is gaining attention (Mahmoudpour et al., 2022); however, consideration of these patient specific factors as part of ICSRs remains under-explored. Crutchley and Keuler examined the impact of differences in the Cytochrome (CYP) enzymes, specifically CYP2D6, on the efficacy of psychotropic drugs and remission rates with comparison among Black, Latinos and Whites and thus provided good support to the addition of genetic information in guiding therapeutic interventions that may minimize risk. Similarly, the articles of Koomdee et al. and Tiwattanon et al. both reported on benefits of the integrating Human Leukocyte Antigen genotyping to reduce possible drug–induced severe cutaneous adverse drug reactions.

The use of machine learning in the pharmacovigilance data mining processes has attracted the interest of many stakeholders (Kreimeyer et al., 2021; Trifirò and Crisafulli, 2022). However, as we advance the use of “*computer algorithms*” to define the patient, we need to remain mindful of ICSR guidelines provided by the International Council on Harmonization, which encourages the inclusion of reporter narratives of cases. Although the unstructured nature of narratives is challenging for aggregated data analysis, such information is crucial for causality assessment. Cai et al. highlighted absence of patient hypersensitivity history in the analysis of cephalosporin induced adverse events in children. The article by Lee et al. reports on a direct relationship between poor medication adherence and adverse events among patients on multiple pharmaceuticals for long-term. Furthermore, the willingness of this patient group to adhere to medication is highly dependent on behavioural factors; which is the focus of the perspective article of Brown et al. and posits for the revision of the ICSR guidelines to support inclusion of such information. These three articles in this special topic serve as a reminder that there is a need for efforts to be made to integrate reported narratives into the signal detection processes.

In conclusion, as recent advances made in pharmacovigilance are poised to improve the science, the contributed articles of this special topic emphasize the need to ensure “the patient” is adequately represented in ICSR. The evidence presented herein add to a topic dearth of information and justify further exploration into the inclusion of more patient related factors in signal detection processes, in order to appropriately attribute blame.
